# The Implications of the Meiotic Spindle Visualization Technique on In Vitro Fertilization (IVF) Outcome in a Perimenopausal Patient

**DOI:** 10.7759/cureus.55375

**Published:** 2024-03-02

**Authors:** Neha Nawale, Jarul Shrivastava, Sanket Mahajan, Shilpa Dutta, Namrata Choudhary, Nandkishor Naik, Akash More

**Affiliations:** 1 Clinical Embryology, School of Allied Health Sciences, Datta Meghe Institute of Higher Education & Research, Wardha, IND; 2 Clinical Embryology, Wardha Test Tube Baby Centre, Datta Meghe Institute of Higher Education & Research, Wardha, IND; 3 Clinical Embryology, Datta Meghe Institute of Higher Education & Research, Wardha, IND; 4 Clinical Embryology, Jaslok Hospital & Research Centre, Mumbai, IND

**Keywords:** intra-uterine insemination (iui), optimal microscope, spindle formation, intra-cytoplasmic sperm injection (icsi), infertility

## Abstract

An infertile couple visited an in vitro fertilization center situated in Maharashtra, India, seeking treatment for primary infertility. The 39-year-old premenopausal woman had a history of two intrauterine inseminations and intracytoplasmic sperm injections (ICSI), along with a history of tuberculosis from six years, and a normal hormonal range. The male was normozoospermic. The patient was given a gonadotropin-releasing hormone antagonist treatment and triggered before 36 hours of ovum pickup (OPU), but the cycle failed. Due to normal blood parameters, it was decided to use an optimal microscope using a polarizing filter to check the timing of meiotic spindle (MS) formation in the oocytes. The patient was triggered again for OPU, and during the procedure, 14 oocytes were retrieved. It was decided to perform ICSI after seven and a half hours of OPU post-observation of MS formation around the same hour. On day 21, the patient was suggested for embryo transfer (ET), where two blastocysts (4AA and 3AA) were transferred into the uterus. After a successful ET, the patient was discharged from the hospital. On day 14, a beta-human chronic gonadotrophin report revealed a positive pregnancy (910 mIU/mL).

## Introduction

WHO defines infertility as a disorder of the reproductive system when, after a year of continuous sexual activity, pregnancy does not occur. Infertility is regarded as a worldwide issue that impacts numerous facets of life for individuals of both genders [1]. Since tuberculosis (TB) is so common in countries like India, infectious disorders that cause infertility have become a significant problem. Consequently, it was important to consider infertility in the local community [2]. Cell division is important for life. It is the process by which a single cell divides into two daughters; each of them keeps the identical genetic blueprint stored within chromosomes due to dependable chromosomal segregation [3]. Oocyte chromosomes align at the meiotic spindle’s (MS) equator during the metaphase of the second meiotic division (MII). This extremely dynamic structure was created by microtubules with one pole connected to the cell cortex situated at the oocyte’s periphery. The first polar body’s (PB1) location did not appear to be an accurate indicator of the MS position. It was expected that the movement of the PB1 rather than the MS was the reason for the angle deviation between the PB1 and the MS [4].

Using a polarized light microscope, researchers discovered numerous components of living tissue, such as bone, muscle, connective tissue, and even the mitotic spindle in dividing cells, were birefringent [5]. Polarized light microscopy (PLM) is a recent, noninvasive method for analyzing human oocytes. As it does not involve cell attachment, the process has no adverse impact on the female gamete. When a cell is illuminated, anisotropic components that result from light polarization can be detected using PLM [6]. Only intracytoplasmic sperm injection (ICSI) can be utilized to study oocytes using PLM since the oocyte’s surrounding cumulus mass is removed during this technique [7]. This case report highlights the utilization of the PolScope visualization technique for MS formation in perimenopausal women seeking ART treatment.

## Case presentation

Patient information

An infertile couple visited our in vitro fertilization (IVF) center in Maharashtra, India. A premenopausal 39-year-old woman and a 40-year-old male came to our infertility clinic, pursuing infertility treatment after experiencing infertility for 12 years in their marriage.

Medical and surgical history

The couple had no history of surgical intervention. The female was suffering from irregular menstrual cycles, occasional pelvic pain, and abnormal vaginal bleeding. She had a medical history of two failed intrauterine inseminations (IUI) and three failed ICSI treatments. The female had a history of genital endotuberculosis six years ago, while the male had no medical history.

Physical examination

The female BMI was 21.2 kg/m^2^, while the male was 24.1 kg/m^2^. A medical examination indicated that both partners were within normal limits.

Investigation

The female underwent a blood test, and the report showed anti-Müllerian hormone 1.2 ng/ml, which was within the normal range, indicating normal ovarian reserve. Also, follicle-stimulating hormone, thyroid-stimulating hormone, luteinizing hormone, and progesterone hormones were within the normal range.

After analyzing the husband’s semen sample, the count was 50 million/ml, motility was 80%, and normal morphology was 6%. The male was normozoospermic.

Treatment

**Figure 1:**
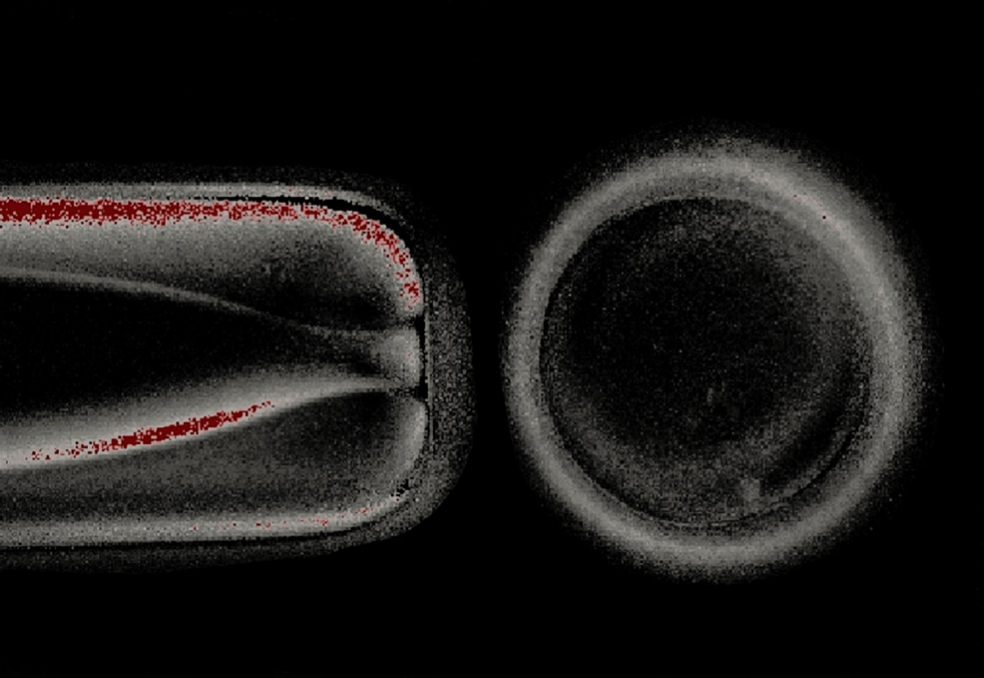
Visualization of oocytes around five hours No presentation of spindle formation was observed.

**Figure 2:**
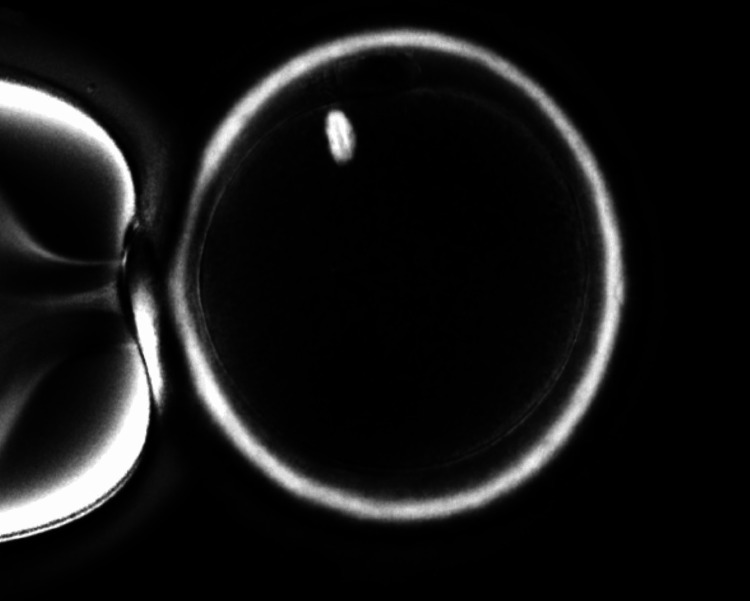
Visualization of oocytes around seven and a half hours The formation of the spindle fiber was observed.

A gonadotropin-releasing hormone antagonist treatment was recommended for the female patient on day two of the menstrual cycle. On day 14, the patient received a subcutaneous injection of 10,000 IU of human chorionic gonadotropin (hCG), which helped in the maturation of oocytes. The patient was recommended for oocyte retrieval after 36 hours of stimulation. During the procedure, 11 oocytes were retrieved: three at the MII stage, five at the metaphase I (MI) stage, and three at the germinal vesicle stage. ICSI was performed with three MII and five MI oocytes on the same day after one hour of ovum pickup (OPU). Five MI oocytes did not fertilize, as confirmed by the absence of two pronuclei; three embryos were formed from MII oocytes, and all were arrested on day two. Later, we examined the blood report of the patient. Due to normal parameters, we suggested the use of an optimal microscope with a polarizing filter to check the time-lapse of forming an MS for the next cycle. Then the patient was stimulated for a second cycle of OPU and triggered before 36 hours of the procedure. Following oocyte retrieval, 14 oocytes were retrieved. After five hours using an optimal microscope, where MS formation of oocytes happened after seven and a half hours post-retrieval, we decided to perform ICSI around the same time. On day one, fertilization was checked, 100% fertilization was observed, and eight good-quality blastocysts were formed on day five. On day 21 of the same menstrual cycle, the patient was advised for embryo transfer (ET), during which two blastocysts (4AA and 3AA) were transferred. Figure 1 denotes that no presentation of spindle formation was observed. Figure 2 denotes the formation of the spindle fiber that was observed.

Follow-up

After a successful ET, the patient was discharged from the hospital. After 14 days of ET, the serum human chorionic gonadotropin (β-hCG) level in a female blood sample tested was 910 mIU/mL. A singleton pregnancy was confirmed through a transvaginal ultrasound. Nuchal translucency and an anomaly scan were found to be normal. The patient was advised to have further follow-up thereafter.

## Discussion

Researchers found that MS visibility was revealed to be an important parameter for oocyte maturity and when to do ICSI in patients over the age of 35 [8]. Our case demonstrated MS visualization as a useful parameter for oocyte maturity and ICSI timing.

Previous studies have shown that spindle birefringence in viable human oocytes could be quantified through morphological analysis and observed with PolScope. In their investigation, the MS’s size was measured using PolScope and categorized into three groups based on the spindle’s cross-sectional area [9]. In our study, we also used PolScope to observe the MS size and morphology of oocytes, but we conducted this study on one patient.

According to Lei et al., PolScope was used to predict chromosomally normal oocytes for insemination and to analyze MSs in IVF [10]. This study indicated the use of a polarized microscope to predict normal chromosomal oocytes and to examine spindle meiotic formation.

It was discovered that there was a considerable correlation between the pregnancy rate and the MS’s light retardancy in human oocytes [11].

Some researchers suggested that a PLM study of the MS in both fresh and frozen oocytes might have helped select eggs that had a high chance of developing into embryos that would lead to pregnancy [6]. In our study, we used PLM to observe the formation of the MS in oocytes.

Konc et al. discovered that the spindle images from live oocytes captured by a PolScope resembled those from fixed oocytes obtained by confocal microscopy. They advised using PolScope spindle images to choose chromosomally normal, high-quality oocytes for IVF [12]. In our case study, images of live oocytes were captured using a PolScope to understand the quality of the oocytes.

## Conclusions

Our case study highlighted the significance of MS visualization in determining oocyte maturity and the optimal time for ICSI, particularly for older patients undergoing infertility treatment. A successful pregnancy resulted from the use of a gonadotropin-releasing hormone antagonist, an ideal microscope equipped with a polarizing filter for MS examination, and a well-planned second OPU. Further studies involving larger patient populations were necessary to validate these findings and improve procedures for individualized infertility treatment plans.
